# Platelet-rich plasma versus hyaluronic acid in the treatment of knee osteoarthritis: a meta-analysis

**DOI:** 10.1186/s13018-020-01919-9

**Published:** 2020-09-11

**Authors:** Jia Zhu Tang, Ming Jun Nie, Jian Zhong Zhao, Guang Cheng Zhang, Qing Zhang, Bo Wang

**Affiliations:** Department of Joint Surgery, Affiliated Hospital of Jiangsu University, Zhenjiang, Jiangsu Province China

**Keywords:** Osteoarthritis, Platelet-rich plasma, Hyaluronic acid, Meta-analysis

## Abstract

**Background:**

This study aimed to evaluate the clinical efficacy of platelet-rich plasma (PRP) injection compared with hyaluronic acid (HA) injection for patients undergoing knee osteoarthritis.

**Methods:**

We systematically searched electronic databases including PubMed, Embase, Web of Science, and the Cochrane Library on January 23, 2020 to identify relevant studies issued in English languages. The outcomes evaluating the efficacy of knee osteoarthritis (KOA) treatment were Western Ontario and McMaster Universities Arthritis Index (WOMAC) scores (WOMAC pain, function, stiffness, and total scores) at 1, 3, 6, and 12 months; International Knee Documentation Committee (IKDC) scores, Lequesne Index score, Visual Analog Scale (VAS) scores, EQ-VAS scores, and KOOS scores. The pooled data were analyzed by Stata 12.0.

**Results:**

A total of 20 RCTs were enrolled in the present meta-analysis. The pooled results demonstrated that platelet-rich plasma (PRP) injection reduced pain more effectively than hyaluronic acid (HA) injection at 6-month and 12-month follow-up evaluated by WOMAC pain scores and VAS scores. EQ-VAS in the patients treated with PRP injection was lower than that in patients with HA injection at 12 months. Moreover, the patients with PRP injection had a better function recovery than those with HA injection at 1-month, 3-month, 6-month, and 12-month follow-up, as evaluated by WOMAC function scores. WOMAC total scores showed significant difference at 6-month and 12-month follow-up. The IKDC scores indicated PRP injection was significantly more effective than HA injection at 3 months and 6 months. However, the Lequesne Index scores, KOOS scores, and adverse events did not show any significant difference between groups.

**Conclusion:**

Intra-articular PRP injection appeared to be more efficacious than HA injection for the treatment of KOA in terms of short-term functional recovery. Moreover, PRP injection was superior to HA injection in terms of long-term pain relief and function improvement. In addition, PRP injection did not increase the risk of adverse events compared to HA injection.

## Background

Knee osteoarthritis (KOA) is a common disease associated with progressive deterioration of the cartilage and narrowing of the joint space [1]. It was reported that KOA in the USA was nearly 27 million, and the number of KOA is continually growing due to the aging population [2, 3].

Patients often advance through multiple treatments to block the progresses; however, there are no therapies proven to alter the progression of KOA development [4]. Current treatments are mainly concentrated on the symptom’s remission with the aim of pain relief and function recovery [5]. Nonsurgical therapies are met with both nonpharmacological and pharmacological approaches [6]. Diet and exercise are the two recommended nonpharmacological treatments but often with poor compliance [7]. Pharmacological treatments for KOA are focused on the administration of oral glucosamine, chondroitin, acetaminophen, celecoxib, glucosamine, and chondroitin [8]. However, the use of NSAIDs and analgesics is often accompanied with side effects [9].

Intra-articular injection, as a minimally invasive therapy, is reported safe and effective for the treatment of KOA [10]. Injections of intra-articular hyaluronic acid (HA) and platelet-rich plasma (PRP) are used as other non-surgical treatment options for the patients with KOA [11]. HA, a high-molecular weight glucosamine, is generated by chondrocytes, synoviocytes, and fibroblasts and responsible for the viscoelasticity and lubrication of the knee joint [12]. It is shown that HA concentrations in osteoarthritic knees have been reduced. Increasing evidences have demonstrated that HA is able to improve joint function, relieve pain, and reduce the dosage of analgesics [13].

Injection of intra-articular HA had been recommended in the management of patients with KOA by the American College of Rheumatology (ACR) in 2012 [14]. PRP is an autologous product derived from patients’ own blood through the process of gradient density centrifugation. PRP contains various growth factors and other bioactive molecules, which may regulate the aberrant inflammatory processes, regenerate tissue structures and thus promote tissue healing [15]. Autologous PRP involves a minimum risk of immune reactions and transmission of infectious diseases, and it has been widely used for the recovery of rotator cuff tendinopathy [16]. Previously, a RCT conducted by Lin et al. [17] revealed that intra-articular injections of leukocyte-poor PRP can provide clinically significant functional improvement for at least 1 year in patients with mild-to-moderate osteoarthritis of the knee.

However, there is still no consensus about which treatment (i.e., PRP vs. HA) is the best possible treatment for knee OA. Di et al. [18] conducted a meta-analysis about PRP versus HA for KOA, and results found that PRP intra-articular injection may be an effective alternative treatment for KOA, though some included studies suggested that the efficacy of PRP was no better than HA. Nevertheless, some studies failed to show PRP providing a superior clinical improvement with respect to HA [19, 20]. This study aimed to compare the efficacy and safety of intra-articular PRP and HA for KOA patients.

## Methods

This systematic review and meta-analysis are performed based on the guidance of the Preferred Reporting Items for Systematic Reviews and Meta-analysis (PRISMA) statement and Cochrane Handbook for Systematic Reviews of Interventions [21]. No ethical approval and patient consent are required because all analyses are based on previous published studies.

### Search strategy and literature selection

We systematically searched electronic databases including PubMed, Embase, Web of Science and the Cochrane Library on January 23, 2020 to identify relevant studies issued in English languages. The search strategy was made for the use of Medical Subject Headings (MeSH) terms and correspondence keywords. The search strategies can be seen in Supplement S1. We also searched relevant reviews and meta-analyses to identify other eligible studies.

Two investigators (Jia Zhu Tang and Ming Jun Nie) independently conducted the initial searches and screened the titles and abstracts for selecting eligible studies. In addition, the reference lists from all the original articles and identified reviews were also manually scanned for additional relevant studies. If either reviewer observed a title or abstract meeting the eligibility criteria, full text of the study was retrieved.

### Inclusion and exclusion

#### Eligibility criteria

The inclusion criteria were as follows:

1. Patients: patients diagnosed with KOA at any grading scale
2. Intervention: intra-articular injection with PRP for intervention
3. Comparison: intra-articular injection with HA for comparison
4. Outcomes: the outcomes concerning efficacy including Western Ontario and McMaster Universities Arthritis Index (WOMAC) scores (WOMAC pain, function, stiffness, and total scores) at 1, 3, 6 and 12 months; International Knee Documentation Committee (IKDC) scores, Lequesne Index scores, Visual Analog Scale (VAS) scores, EQ-VAS scores, Knee Injury and Osteoarthritis Outcome Scores (KOOS); the outcomes concerning safety including postoperative adverse events (pain, stiffness, dizziness, febrile syndrome, headache, flu, or infection). WOMAC scores were identified as the primary outcome due to the comprehensive reaction knee joint function.
5. Studies: only randomized controlled clinical trials were included

The exclusion criteria were as follows:

1. Patients who suffered from bilateral KOA
2. Nonrandomized studies
3. Articles that which we were unable to obtain the relevant data for pooled analysis.

### Data extraction

Two reviewers independently extracted data from each study using a standardized data extraction form. Disagreements were resolved by discussion, and those unresolved through discussion were reviewed by a third reviewer. The following variables were included: first author, publication year, country, number of participants, age, sex, body mass index, radiographic classification, and follow-up. Moreover, we collected injection doses, times, and intervals of PRP and HA injections. Primary outcomes included WOMAC pain and WOMAC function scores at 1, 3, 6, and 12 months. Secondary outcomes were WOMAC total scores, WOMAC stiffness scores, IKDC scores, VAS scores, EQ-VAS scores, KOOSs, Lequesne Index scores, and adverse events. We intended to contact authors for detail information when the reported data were inadequate.

### Risk of bias assessment

Two reviewers (Jian Zhong Zhao and Guang Cheng Zhang) independently evaluated the risk of bias of each RCT by the Cochrane Risk of Bias tool. Each article was assessed based on the following seven items: random sequence generation (selection bias), allocation concealment (selection bias), blinding of participants and personnel (performance bias), blinding of outcome assessment (detection bias), incomplete outcome data (attrition bias), selective reporting (reporting bias), and other bias. Every item was scored as high, low, or unclear. Any discrepancies shall be settled by consensus between the two reviewers. If necessary, a third reviewer shall be consulted (Bo Wang).

### Statistical analysis

As for outcomes measurements (WOMAC pain, function, and total scores; IKDC scores; and Lequesne scores) in each study, the mean difference between the baseline and post intervention was calculated to compare the efficacy of intervention with the control group. The present study was conducted by using Stata 12.0 (Stata Corp., College Station, TX, USA) for meta-analysis. The standard mean difference (SMD) and 95% confidence interval (CI) were calculated for continuous outcomes (WOMAC pain, function, stiffness, and total scores; IKDC scores; VAS scores; EQ-VAS scores; KOOSs; and Lequesne scores), while risk ratios (RRs) with 95% CIs were adopted for dichotomous outcome (adverse events). Heterogeneity among studies was accessed by using the I^2^ statistic. The I^2^ values of 25%, 50%, and 75% were respectively considered to be the cut-off points for low, moderate, and high heterogeneity. Random effects method was used for all meta-analyses. *P* value < 0.05 was considered to be of statistical significance.

## Results

### Literature search

**Fig. 1:**
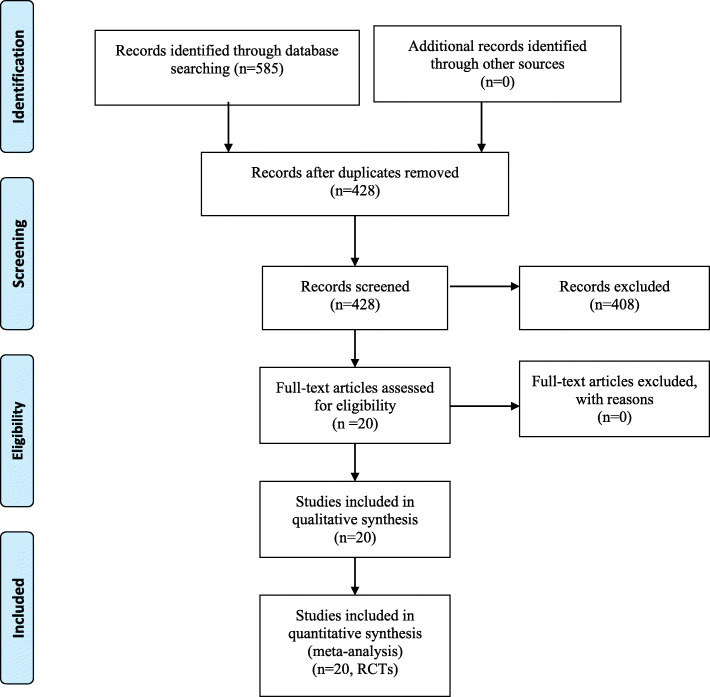
Flowchart of the study search and inclusion criteria.

Figure 1 shows the details of the literature search. A total of 190 records were identified as potentially relevant studies. By removing duplicates, scanning titles, and reading abstracts, 83 full-text articles were assessed for eligibility. Ultimately, 19 RCTs [17, 19, 20, 22–38] were included for data extraction and meta-analysis.

### Study characteristics

The study characteristics are available in Table 1. These studies were published from 2011 to 2019 with a total of 1281 patients. The sample size of the studies ranged from 10 to 104. There were 654 patients in the PRP injection group and 627 patients in the HA injection group. The follow-up period ranged from 3 to 18 months. The demographic features between the two groups in each study were similar. The administrated timing and dosage of PRP and HA injections are shown in Table 2, which were varied among these studies.

**Table 1:** General characteristic of the included studies

Author	Country	No. of patients (PRP vs HA)	Age (years, PRP vs HA)	Sex (male, PRP vs HA)	BMI (PRP vs HA)	Radiographic classification (PRP vs HA)					Follow-up (m)
						0	I	II	III	IV	
Ahmad 2018 [22]	Egypt	45/44	56.2/56.8	14/14	NS	0/0	8/7	17/19	20/18	0/0	6
Buendia-Lopez 2018 [23]	Spain	33/32	56.2/56.6	16/15	24.9/24.9	0/0	18/18	15/14	0/0	0/0	12
Cerza 2012 [24]	Italy	60/60	66.5/66.2	25/28	NS	0/0	21/25	24/22	15/13	0/0	6
Cole 2017 [20]	USA	49/50	55.9/56.8	28/20	27.4/29	0/0	3/0	26/27	20/22	0/0	12
Di Martino 2019 [25]	Italy	85/82	52.7/57.5	53/47	27.2/26.8	NS					60
Duymus 2017 [26]	Turkey	33/34	60.4/60.3	1/1	27.6/28.4	0/0	0/0	22/24	21/10	11/0	12
Filardo 2015 [19]	Italy	94/89	53.3/57.6	60/52	26.6/26.9	NS	NS	NS	NS	NS	12
Gormeli 2017 [27]	Turkey	39/39	53.7/53.5	16/17	28.7/29.7	0/0	26/30			13/14	6
Huang 2019 [28]	China	40/40	54.5/54.8	25/19	25.2/24.5	0/0	40/40		0/0	0/0	12
Li 2011 [29]	China	15/15	57.6/58.2	6/7	24.3/21.0	0/0	6/6	2/3	4/3	3/3	6
Lin 2019 [17]	China	31/29	61.2/62.5	9/10	24.0/26.3	5/6^#^	16/14^#^	10/9^#^			12
Louis 2018 [30]	France	24/24	53.2/48.5	14/11	25.6/27.0	0/0	0/0	24/24			6
Montanez-Heredia 2016 [31]	Spain	27/26	66.3/61.5	12/9	29.0/30.4	0/0	5/2	10/9	12/15	0/0	6
Paterson 2016 [32]	Australia	11/10	49.9/52.7	8/7	27.9/28.9	0/0	0/0	11/10		0/0	3
Raeissadat 2015 [33]	Iran	77/62	56.9/61.1	8/15	28.2/27.0	0/0	5/0	34/29	29/23	9/10	12
Sanchez 2012 [34]	Spain	89/87	60.5/58.9	43/42	27.9/28.2	45/43^#^	32/33^#^	12/11^#^			6
Su 2018 [35]	China	25/30	54.2/53.1	11/12	28.2/28.7	0/0	0/0	16/13	11/12	0/0	6
Vaquerizo 2013 [36]	Spain	48/48	62.4/64.8	16/22	30.7/31.0	NS	NS				12
Yu 2018 [37]	China	104/88	46.2/51.5	50/48	NS	NS	NS				12
Tavassoli 2019 [38]	Iran	28/27	63.2/66.0	5/8	28.4/28.9		21/22	35/32			3

*NS* not stated, *PRP* platelet-rich plasma, *HA* hyaluronic acid

^#^ Ahlbäck grade, and the rest were K–L grades

**Table 2:** Detail treatment protocols of PRP and HA injections

Author	PRP				HA		
	Injection dose (ml)	Times	Intervals	Type	Injection dose (ml)	Times	Intervals
Ahmad 2018 [22]	4	3	2 Weeks	LRP	20 mg/2 ml high	3	2 Weeks
Buendia-Lopez 2018 [23]	5	NS	NS	LPP	60 mg/2 ml high	NS	NS
Cerza 2012 [24]	5.5	4	Weekly	LPP	20 mg/2 ml high	4	Weekly
Cole 2017 [20]	4	3	Weekly	LPP	16 mg/2 ml high (6 MDa)	3	Weekly
Di Martino 2019 [25]	5	3	Weekly	LRP	30 mg/2 ml high (> 1500 kDa)	3	Weekly
Duymus 2017 [26]	5	2	Monthly	LRP	40 mg/2 ml high (1600 kDa)	1	Monthly
Filardo 2015 [19]	5	3	Weekly	LRP	30 mg/2 ml high (> 1500 kDa)	3	Weekly
Gormeli 2017 [27]	5	3	Weekly	LRP	30 mg/2 ml	3	Weekly
Huang 2019 [28]	2	3	Weekly	LPP	4 ml (500–730 kDa)	3	Weekly
Li 2011 [29]	3.5	3	3 Weeks	LRP	2 ml	3	3 Weeks
Lin 2019	5	3	Weekly	LPP	20 mg/2 ml	3	Weekly
Louis 2018 [30]	3	1	NS	LPP	60 mg/3 ml	1	NS
Montanez-Heredia 2016 [31]	NS	3	2 Weeks	LPP	NS	3	2 Weeks
Paterson 2016 [32]	3	3	Weekly	LRP	3 ml	3	Weekly
Raeissadat 2015 [33]	43,927	2	Monthly	LRP	20 mg/2 ml (500–730 kDa)	3	Weekly
Sanchez 2012 [34]	8	3	Weekly	LPP	NS	3	Weekly
Su 2018 [35]	6	2	2 Weeks	LRP	2 ml	5	Weekly
Vaquerizo 2013 [36]	8	3	2 Weeks	LPP	NS	1	NS
Yu 2018 [37]	2–14	4	Weekly	LRP	0.1–0.3 mg	4	Weekly
Tavassoli 2019 [38]	4–6	4	3 Weeks	LRP	30 mg/2 ml (500–730 kDa)	4	Weekly

### Risk of bias

**Fig. 2:**
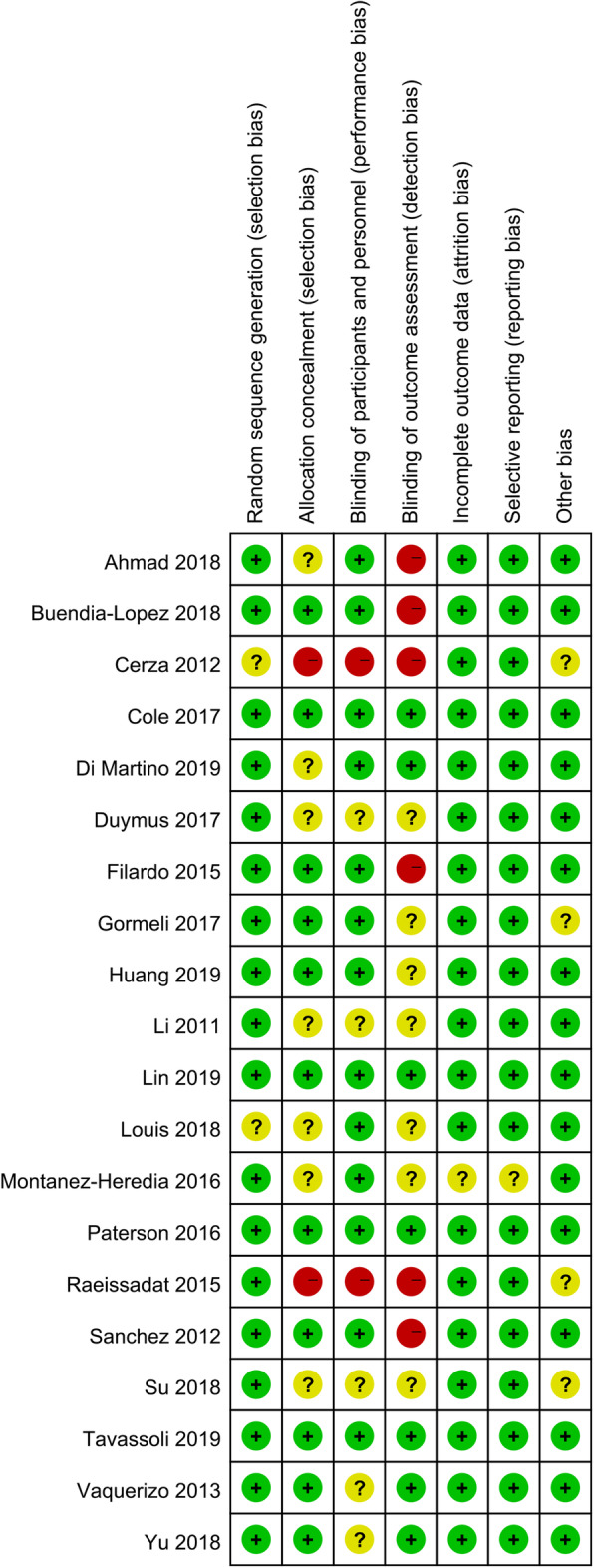
Risk of bias summary of included in randomized controlled trials. 1, no bias; –, bias; ?, bias unknown

**Fig. 3:**
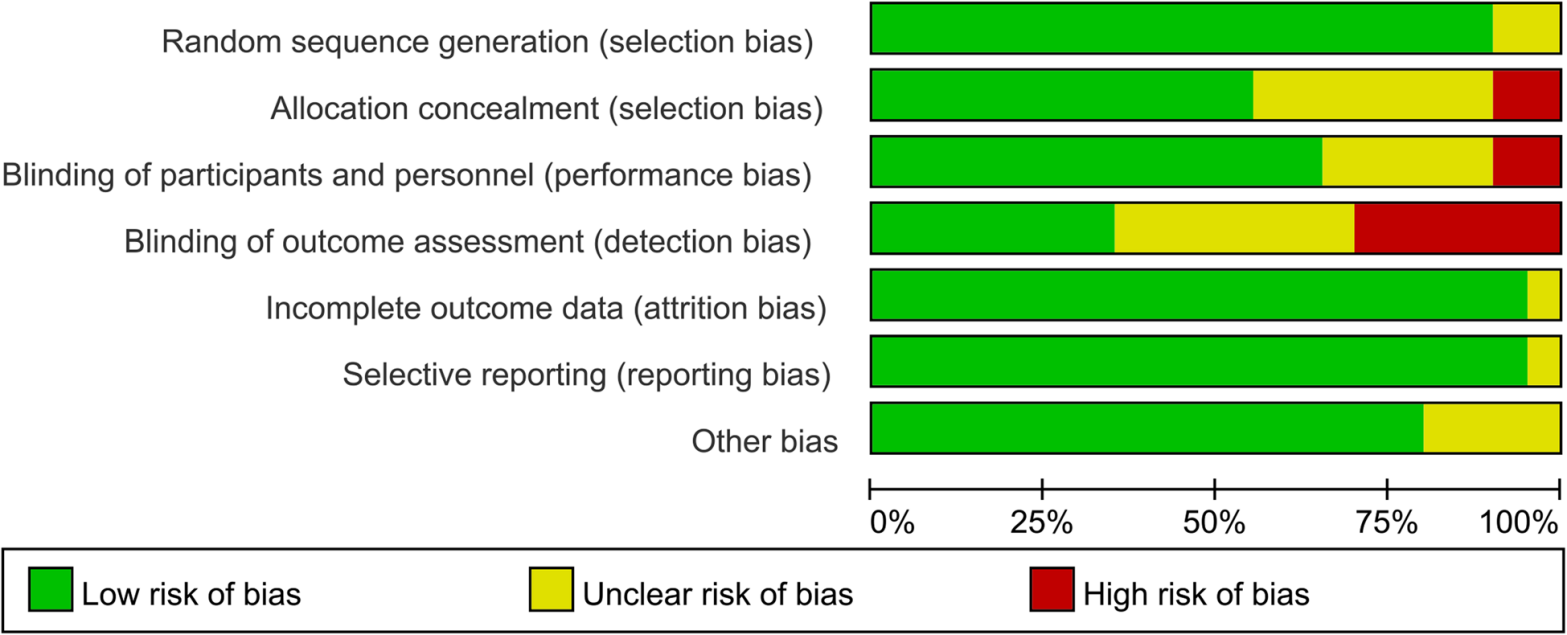
Risk of bias graph in randomized controlled trials

Figures 2 and 3 reveal the risk of bias summary and graph of all included RCTs. Among the twenty RCTs, random sequence generation were recorded adequately in eighteen studies [17, 19, 20, 22, 23, 25–28, 31–39] and unclear in two studies [24, 30]. Allocation concealment were recorded adequately in eleven studies [17, 19, 20, 23, 27, 28, 32, 34, 36–38], unclear in seven studies [22, 25, 26, 30, 35, 39], and high in two studies [24, 33]. The performance bias was recorded adequately in thirteen studies [17, 19, 20, 23, 27, 28, 30–32, 34, 38], unclear in five study [26, 35–37, 39], and high in two studies [24, 33]. Only four studies [24, 27, 33, 35] reveal unclear risk of bias for other bias.

### Outcomes of the meta-analysis

#### WOMAC total scores

**Fig. 4:**
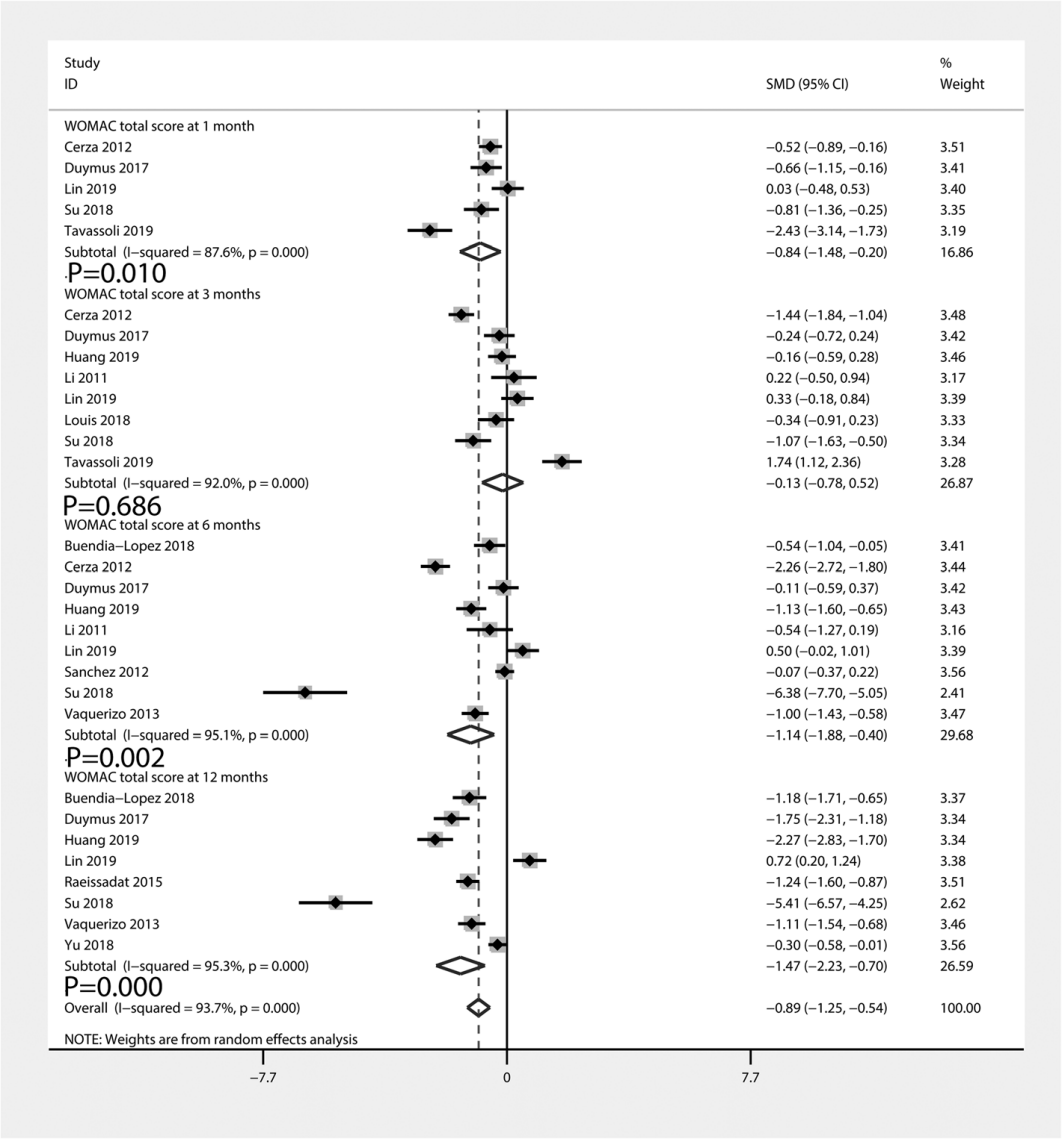
Forest plot for WOMAC total scores between PRP and HA groups

Figure 4 summarizes the WOMAC total scores comparing PRP injection with HA injection. A total of five [17, 24, 26, 35, 38], eight [17, 24, 26, 28–30, 35, 38], nine [17, 23, 24, 26, 28, 29, 34–36], and eight [17, 23, 26, 28, 33, 35–37] studies reported the WOMAC total scores at 1, 3, 6, and 12 months, respectively. The pooled data indicated that, compared with the HA group, PRP injection was associated with a decrease of the WOMAC total scores at 1 month (SMD = − 0.84, 95% CI − 1.48 to − 0.20, *P* = 0.010), 6 months (SMD = − 1.14, 95% CI − 1.88 to − 0.40, *P* = 0.002), and 12 months (SMD = − 1.47, 95% CI − 2.23 to − 0.70, *P* = 0.000). Nevertheless, there was no statistically significant difference between PRP and HA injections at 3 months (SMD = − 0.13, 95% CI − 0.78 to 0.52, *P* = 0.686). Heterogeneity was significant among these pooled results (*I*^2^ = 87.6%, 92.0%, 95.1%, and 95.3%, respectively).

#### WOMAC function scores

**Fig. 5:**
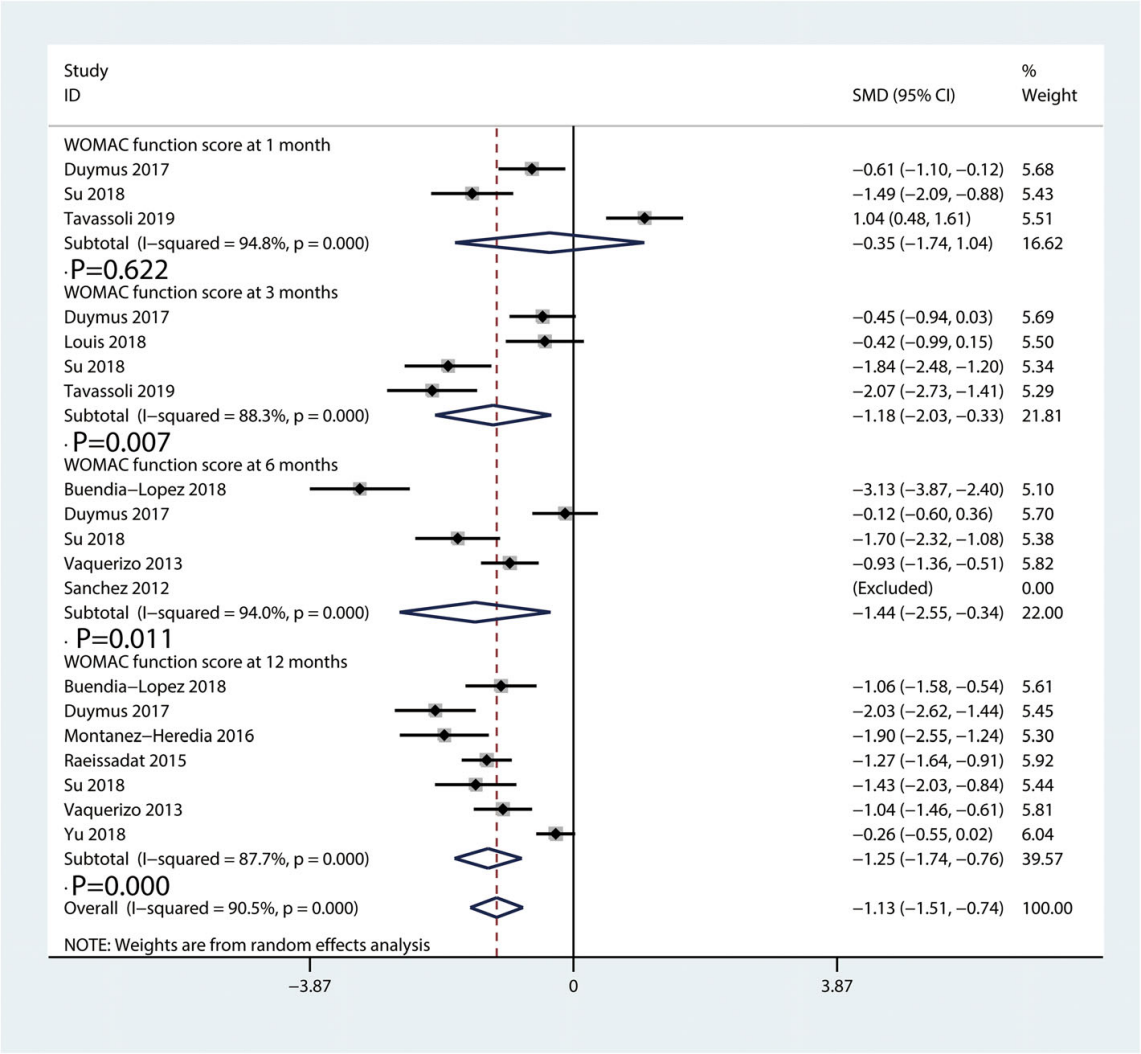
Forest plot for WOMAC function scores between PRP and HA groups

The WOMAC function scores comparing PRP injection with HA injection are available in Fig. 5. A total of three [26, 35, 38], four [26, 30, 35, 38], five [23, 26, 34–36], and seven [23, 26, 31, 33, 35–37] studies described the function recovery evaluated by the WOMAC function scores at 1, 3, 6, and 12 months, respectively. The pooled results revealed PRP injection provided a better function recovery than HA injection at 3 (SMD = − 1.18, 95% CI − 2.03 to − 0.33, *P* = 0.007), 6 (SMD = − 1.44, 95% CI − 2.55 to − 0.34, *P* = 0.011), and 12 months (SMD = − 1.25, 95% CI − 1.74 to − 0.76, *P* = 0.000). There was no statistically significant difference between PRP and HA injections for the WOMAC function scores at 1 month (SMD = − 0.35, 95% CI − 1.74 to 1.04, *P* = 0.622).

#### WOMAC stiffness scores

**Fig. 6:**
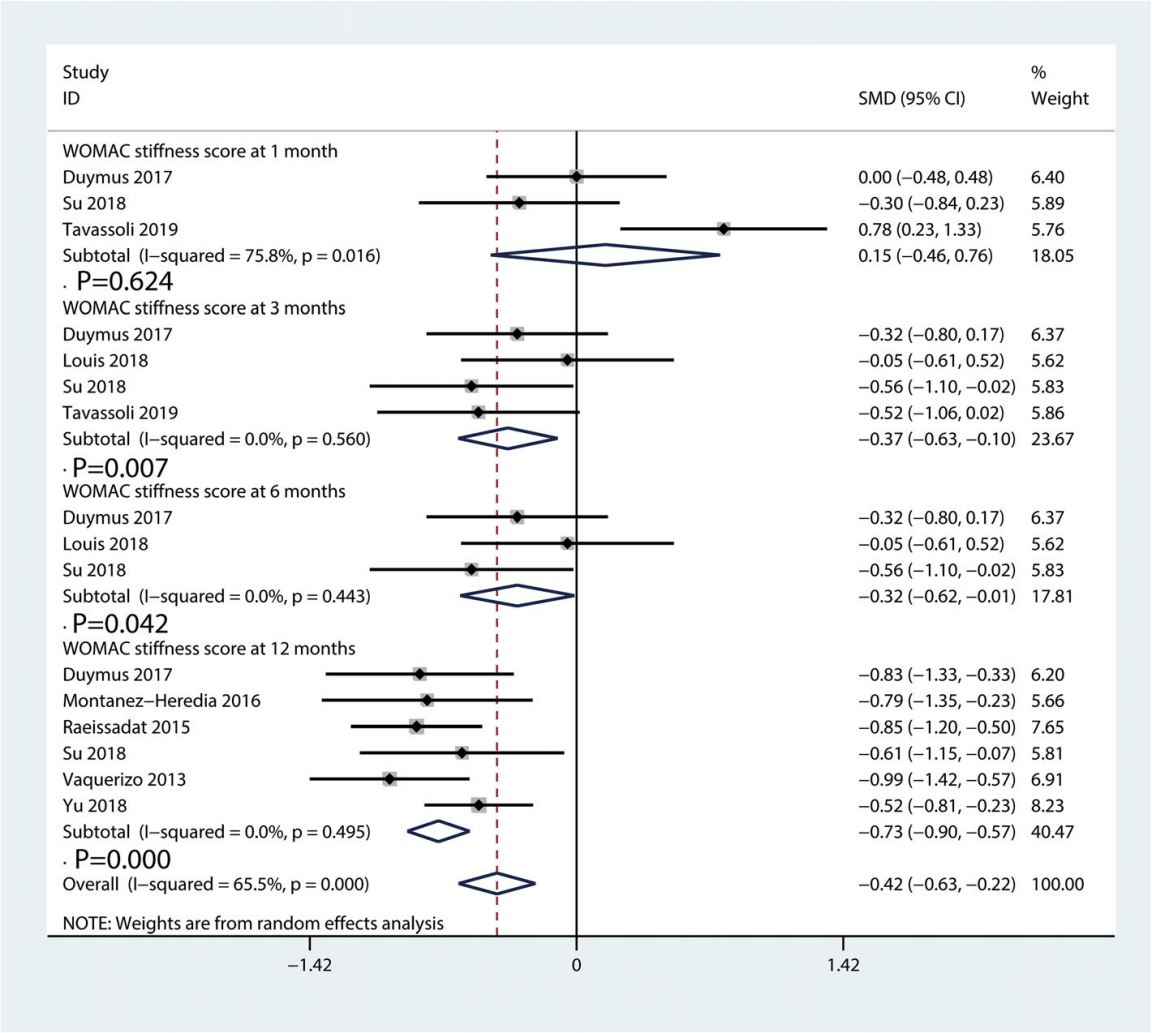
Forest plot for WOMAC stiffness scores between PRP and HA groups

Figure 6 presents the WOMAC stiffness scores comparing PRP injection with HA injection. A total of three [26, 35, 38], four [26, 30, 35, 38], three [26, 30, 35], and six [26, 35–37] studies showed the WOMAC stiffness scores at 1, 3, 6, and 12 months, respectively. PRP injection showed more effective than HA injection in improving knee stiffness at 3 (SMD = − 0.37, 95% CI − 0.63 to − 0.10, *P* = 0.007, Fig. 6), 6 (SMD = − 0.32, 95% CI − 0.62 to − 0.01; *P* = 0.042, Fig. 6) and 12 months (SMD = − 0.73, 95% CI − 0.90 to − 0.57, *P* = 0.000, Fig. 6). However, the pooled results showed that there was no significant difference in knee stiffness improvement at 1 month (SMD = 0.15, 95% CI − 0.46 to 0.76, *P* = 0.624, Fig. 6) between the PRP and HA injection groups.

#### WOMAC pain scores

**Fig. 7:**
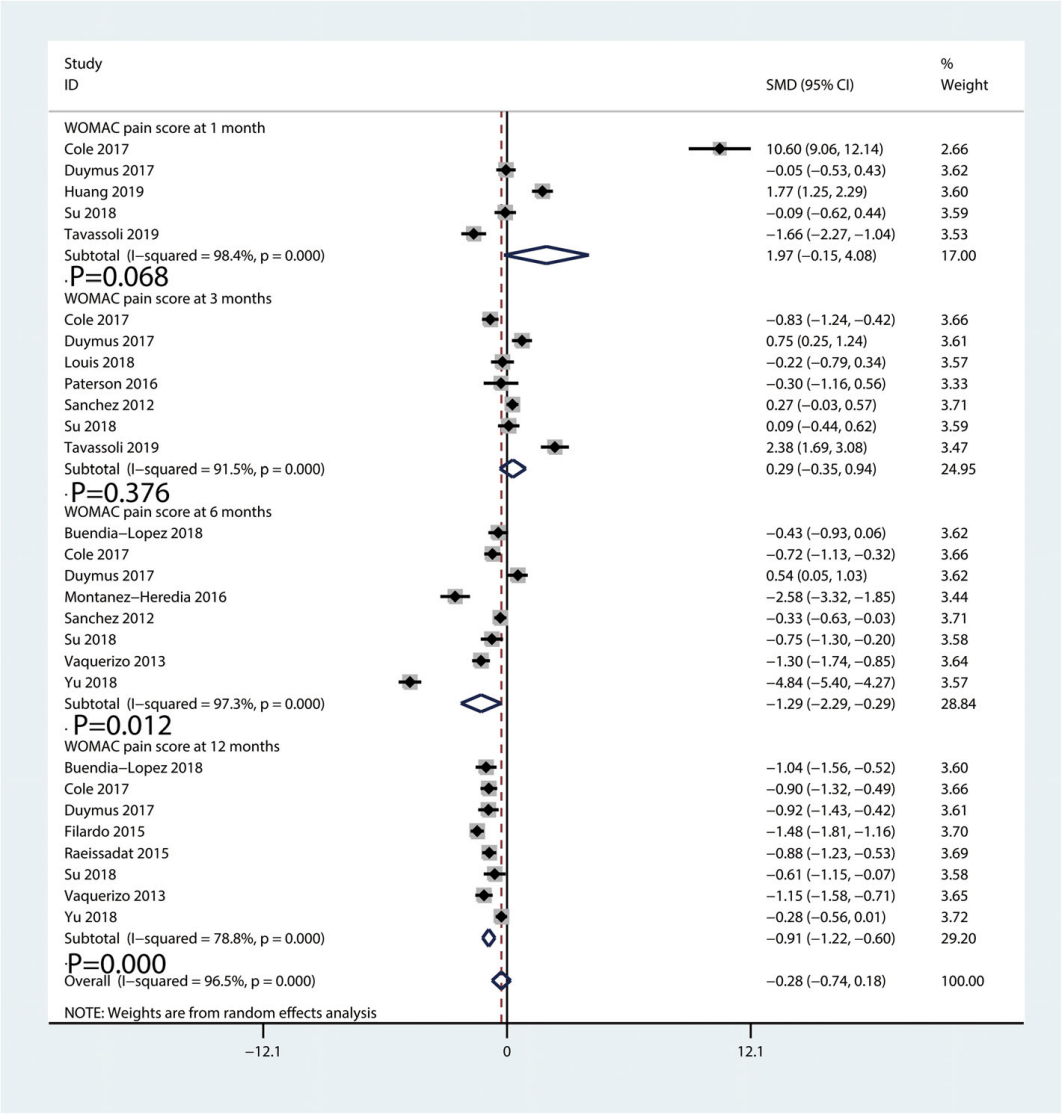
Forest plot for WOMAC pain scores between PRP and HA groups

The WOMAC pain scores comparing PRP injection with HA injection is shown in Fig. 7. A total of five [20, 26, 28, 35, 38], seven [20, 26, 30, 32, 34, 35, 38], eight [20, 23, 26, 31, 34–37], and eight [19, 20, 23, 26, 33, 35–37] studies investigated the WOMAC pain scores at 1, 3, 6, and 12 months, respectively. The pooled results revealed patients with PRP injection got better improvement than those with HA injection at 6 (SMD = − 1.29, 95% CI − 2.29 to − 0.29, *P* = 0.012, Fig. 7) and12 months (SMD = − 0.91, 95% CI − 1.22 to − 0.60, *P* = 0.000, Fig. 7).

There was no significant difference between PRP and HA at 1 month (SMD = 1.97, 95% CI − 0.15 to 4.08, *P* = 0.068, Fig. 7) and 3 months (SMD = 0.29, 95% CI − 0.35 to 0.94, *P* = 0.376, Fig. 7).

#### VAS scores

**Fig. 8:**
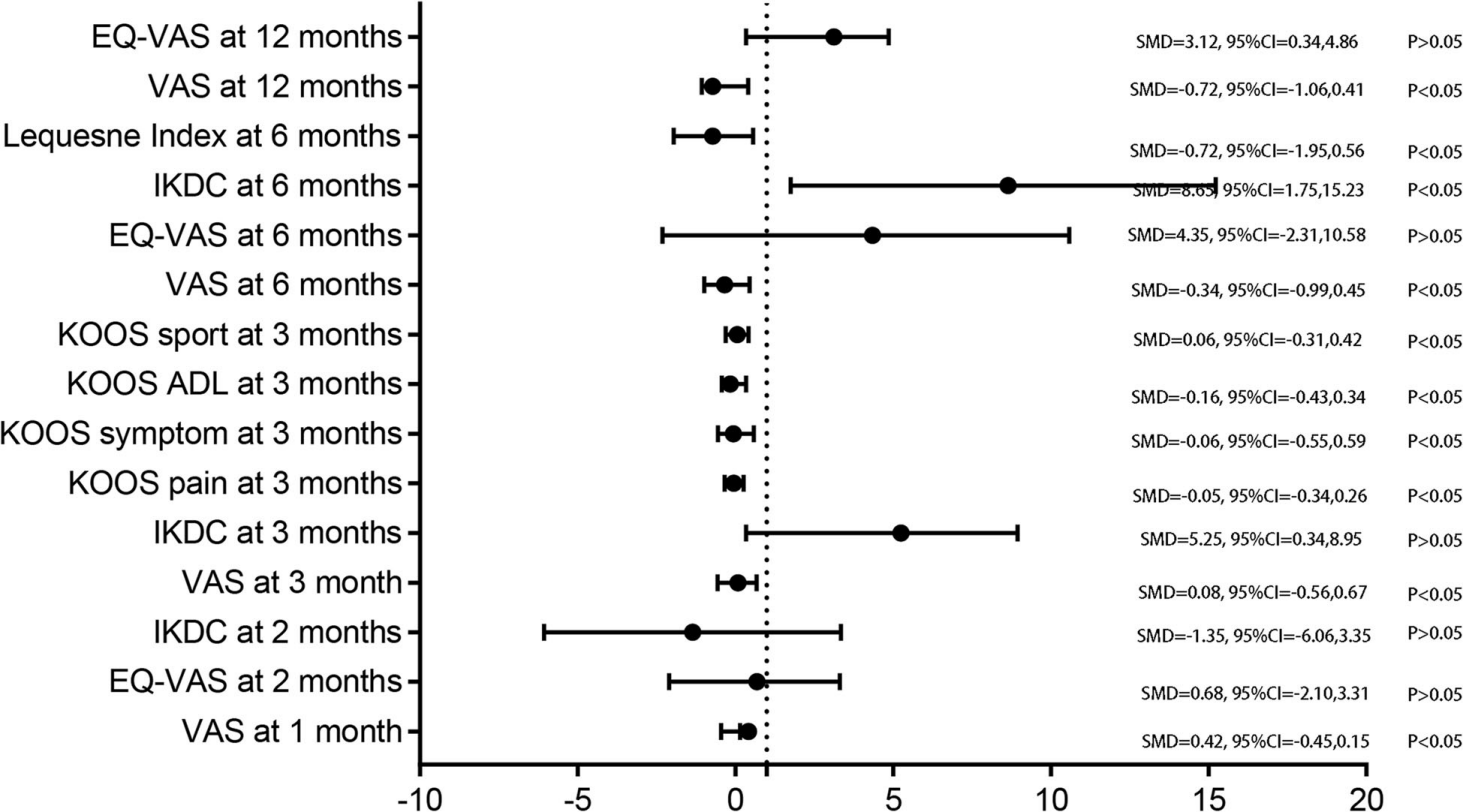
Comprehensive display of the outcomes for VAS scores, IKDC scores, Lequesne index score, EQ-VAS scores, and KOOSs between PRP and HA groups

Figure 8 shows the VAS scores comparing PRP injection with HA injection. The patients with PRP injection had better pain relief than those with HA injection at 1 month (SMD = 0.42, 95% CI − 0.45 to 0.15, *P* < 0.05), 3 months (SMD = 0.08, 95% CI − 0.56 to 0.67, *P* < 0.05), 6 months (SMD = − 0.34, 95% CI − 0.99 to 0.45, *P* < 0.05), and 12 months (SMD = − 0.72, 95%CI − 1.06 to 0.41, *P* < 0.05).

#### IKDC scores

The IKDC scores comparing PRP injection with HA injection are available in Fig. 8. PRP injection was more effective than HA injection at 6 months (SMD = 8.65, 95% CI 1.75 to 15.23, *P* < 0.05, Fig. 8). However, the pooled data suggested there was no significant discrepancy comparing PRP injection with HA injection at 2 months (SMD = − 1.35, 95% CI − 6.06 to 3.35, *P* > 0.05, Fig. 8) and 3 months (SMD = 5.25, 95% CI 0.34 to 8.95, *P* > 0.05, Fig. 8).

#### Lequesne Index scores

The Lequesne Index scores comparing PRP injection with HA injection are available in Fig. 7. PRP injection was more effective than HA injection for the Lequesne Index scores at 6 months (SMD = − 0.72, 95% CI − 1.95 to 0.56, *P* < 0.05, Fig. 8).

#### EQ-VAS scores

The pooled data did not find any significant difference between PRP injection and HA injection at 2 months (SMD = 0.68, 95% CI − 2.10 to 3.31, *P* > 0.05, Fig. 8), 6 months (SMD = 4.35, 95% CI − 2.31 to 10.58, *P* > 0.05, Fig. 8) and 12 months (SMD = 3.12, 95% CI 0.34 to 4.86, *P* > 0.05, Fig. 8).

#### KOOSs

The KOOSs comparing PRP injection with HA injection are presented in Fig. 8. The pooled analysis demonstrated that PRP was associated with a reduction of the symptom (SMD = − 0.06, 95% CI − 0.55 to 0.59, *P* < 0.05, Fig. 8), pain (SMD = − 0.05, 95% CI − 0.34 to 0.26, *P* < 0.05, Fig. 8), activities of daily life (SMD = − 0.16, 95% CI − 0.43 to 0.34, *P* < 0.05, Fig. 8) and sport (SMD = − 0.06, 95% CI − 0.31 to 0.42, *P* < 0.05, Fig. 8).

#### Adverse events

**Fig. 9:**
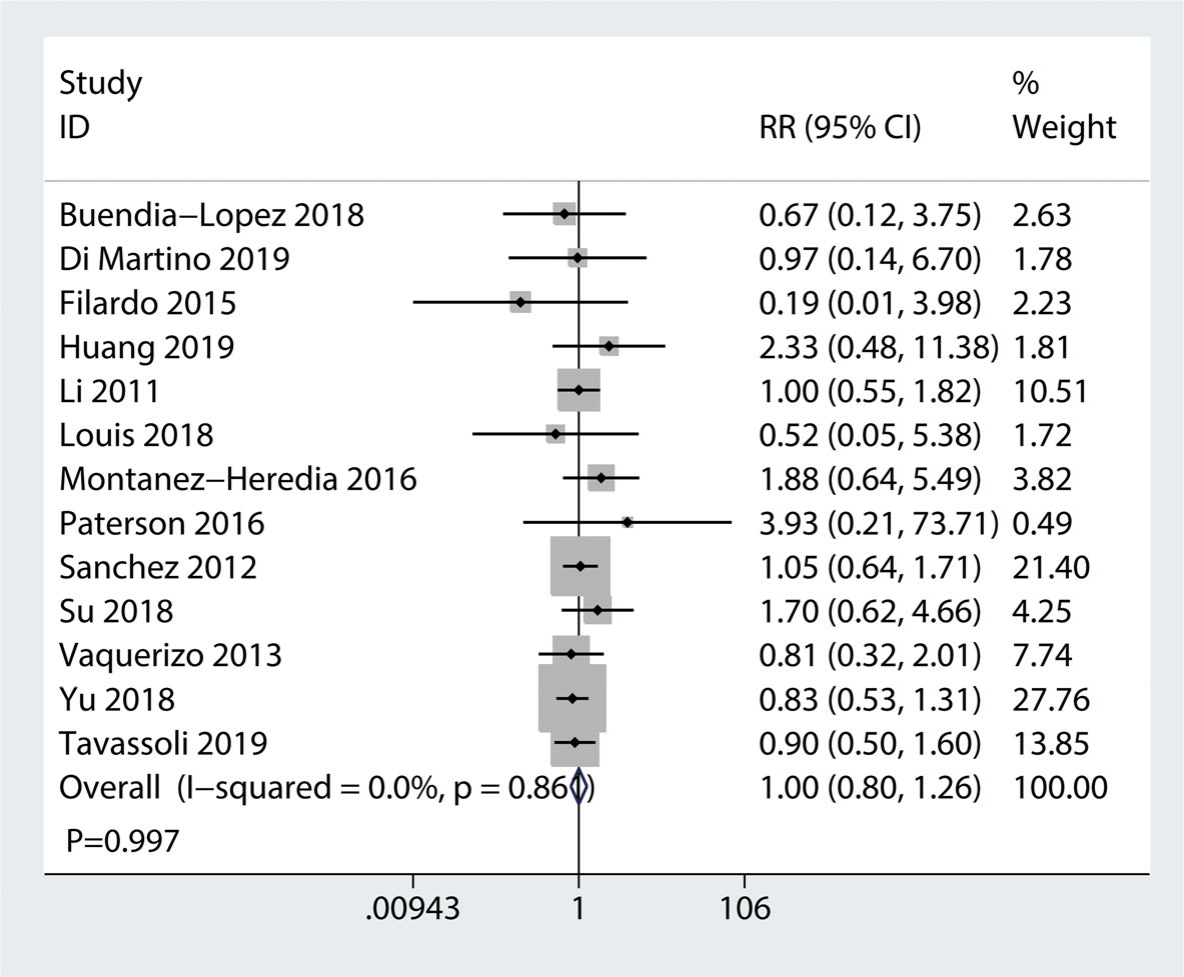
Forest plot for adverse events between PRP and HA groups

Thirteen studies with a total of 1281 patients reported the incidence of advance events comparing PRP injection with HA injection advance events (Fig. 9). The pooled results demonstrated that there was no significant difference between PRP injection and HA injection (RR = 1.00, 95% CI 0.80 to 1.26, *P* = 0.997), with no heterogeneity (*I*^2^ = 0%).

### Subgroup analyses

Table 3 shows the results of subgroup analyses for the WOMAC total scores at 12 months. The subgroup analyses based on the number of PRP injections, PRP spinning approach (single or double), PRP classification (LP-PRP or LR-PRP), fresh or frozen PRP, whether an activator was used, and risk of bias (low or unclear/high) were conducted for exploring the WOMAC total scores at 12 months.

**Table 3:** Subgroup analyses of PRP compared with HA for WOMAC total scores at 12 months

Subgroup	No. of trials	Standard mean difference (95% CI)	*P* value	*I*^2^ (%)	Test of interaction, *P*
Total	8	− 1.47 (− 2.23, − 0.70)	0.000	95.3	
Number of PRP injections					
1	4	− 1.15 (− 2.14, − 0.85)	0.000	69.3	0.006
≥2	4	− 1.68 (− 2.35, − 0.72)	0.000	98.2	
PRP spinning approach					
Single	3	− 1.13 (− 2.22, − 0.85)	0.000	0.0	0.028
Double	5	− 1.62 (− 2.34, − 1.11)	0.000	88.4	
PRP classification					
LPP	3	− 2.89 (− 6.55, − 0.68)	0.000	82.4	0.002
LRP	5	0.86 (0.15, 1.56)	0.000	83.5	
Fresh or frozen PRP					
Fresh	5	0.78 (0.54,1.01)	0.000	57.8	0.105
Frozen	3	0.50 (0.33,0.67)	0.000	88.1	
Activator used					
Yes	4	− 2.78 (− 4.32, − 1.15)		82.1	0.031
No	4	0.58 (0.12, 1.58)		93.4	
Risk of bias					
Low	6	− 1.23 (− 2.08, − 0.35)	0.002	85.9	0.254
Unclear/high	2	− 1.33 (− 2.24, − 0.46)	0.004	89.4	
Molecular weight					
High	4	− 1.90 (− 4.53, 0.73)	0.157	97.9	0.003
Low	4	− 1.31 (− 1.97, − 0.64)	0.000	92.2	
Structure					
Cross-linked	4	− 2.06 (− 3.20, − 0.92)	0.352	96.2	0.000
Not cross-linked or unclear	4	− 0.57 (− 1.78, 0.63)	0.000	95.0	

The findings of WOMAC pain, stiffness, function, and VAS at 12 months were consistent in all subgroup analyses except for the number of PRP injections, PRP single spinning approach, LP PRP, without using an activator, molecular weight, and structure subgroups. In the subgroups of ≥ 2 injections, double spinning approach, LP-PRP, and activator use, we found that PRP was associated with significantly better WOMAC than HA at 12 months (Table 3).

In the subgroup analyses dividing into low and high molecular weight groups, results showed a PRP significant reduction in WOMAC at 12 months than HA with low molecular weight (SMD − 1.31, 95% CI − 1.97 to − 0.64], *P* = 0.000, *I*^2^ = 92.2%, Table 3), but not in HA with high molecular weight (SMD − 1.90, 95% CI = − 4.53 to 0.73, *P* = 0.157, *I*^2^ = 97.9%**,** Table 3). Moreover, there was a PRP significant reduction in WOMAC at 12 months than HA with no cross-link or unclear (SMD − 0.57, 95% CI − 1.78 to 0.63, *P* = 0.000, *I*^2^ = 95.0%, Table 3), but not in HA with cross-link (SMD − 2.06, 95% CI = − 3.20 to − 0.92, *P* = 0.352, *I*^2^ = 96.2%, Table 3).

## Discussion

### Main finding

The pooled results showed that intra-articular PRP injection appeared to be more efficacious than HA injection for the treatment of KOA in terms of short-term functional recovery. Moreover, PRP injection was superior to HA injection in terms of long-term pain relief and function improvement. In addition, PRP injection did not increase the risk of adverse events when compared with HA injection. The level of evidence, which was undermined by heterogeneity and/or study design limitations, was moderate or low, indicating that the degree of benefit must be studied although the benefit is conclusive. PRP is an autologous concentrate of human platelets isolated through centrifugation of the patient’s blood, containing numerous components containing variety of growth factors, cytokines, and many other bioactive proteins [40].

Based on preclinical research, it is known that PRP ameliorates the degeneration of cartilage by stimulation of mesenchymal stem cell migration, proliferation, and differentiation into articular chondrocytes. PRP affects the progression of KOA via inhibition of inflammatory cytokines and altering the level of enzymatic expression and thus promotes cartilage repair [41]. Moreover, several clinical trials and systematic reviews have demonstrated that PRP have the ability to relive osteoarthritic symptoms up to 12 months postinjection, including pain, stiffness, and function failure [42].

HA is the most important component of articular fluid and responsible for the viscoelastic and lubricant capabilities in joints [43]. It is involved in chondroprotection, proteoglycan, and glycosaminoglycan synthesis as well as anti-inflammation. In addition, intra-articular HA injection can significantly reduce the apoptosis rates of chondrocytes [44]. Clinical researches have shown HA injection in patients with KOA has the potential to reduce knee pain, improve function, and quality of life [39].

Interestingly, numerous studies have focused on the clinical efficacy between PRP and HA in the KOA treatment. Duymus et al. [26] compared the efficacy of intra-articular injections of PRP with HA for KOA treatment. They found that PRP injection was more successful than HA injection in the treatment of mild–moderate knee OA. PRP application could provide at least 12 months of pain-free daily living activities. Similarly, Lin et al. [17] investigated the discrepancy between PRP and HA in therapy of KOA and suggested that intra-articular injections of leukocyte-poor PRP (LP-PRP) improved function recovery for at least 1 year in patients with mild-to-moderate osteoarthritis of the knee. Furthermore, Ahmad et al. explored the clinical outcomes of PRP injection with changes in the ultrasonography structural appearance [22]. They observed that intra-articular injections of PRP were associated with improved synovial hypertrophy and vascularity scores and less effusion. However, PRP injection failed to perform better efficacy than HA injection in several clinical studies. Filardo et al. [19] found that the patients with PRP injection could not obtain a better clinical outcome than those treated with HA injection. With a long-term follow-up of 5 years, Di Martino et al. [25] concluded that PRP injection did not provide an overall superior clinical improvement compared with HA injection in terms of functional improvement at any follow-up point. Although LP-PRP injection showed more effective in terms of clinical improvement with respect to HA injection, there was no influence on the X-ray and MRI performance of cartilage progression at 52 weeks follow-up.

Therefore, it still remains a contradiction whether PRP injection is superior to HA injection in the treatment of KOA. Previous systematic review and meta-analysis also evaluated the efficacy of PRP injection compared with HA injection in the treatment of KOA. Laudy et al. [45] enrolled 10 trials and found that PRP injection performed better clinical outcomes than HA injection on pain reduction at 6 months postinjection. Recent meta-analysis by Han et al. [44] pooled 14 RCTs and suggested that PRP injection might be more effective with respect to HA injection in terms of long-term pain relief and functional improvement. The biggest flaw of this meta-analysis was that they included bilateral knee OA, and thus, a large clinical heterogeneity existed. However, Zhang et al. [13] analyzed 13 studies (10 RCTs and 3 non-randomized studies) and concluded that PRP injection was not obviously superior to HA in KOA. Pooled RCTs and non-RCTs for meta-analysis violates the PRISMA guideline for meta-analysis, and selection bias is ineluctable. The present study is, to our knowledge, the most comprehensive, up-to-date, and with the largest sample size (*n* = 19; totally, 1281 patients) meta-analysis undertaken to estimate the efficacy and safety of PRP versus HA in OA.

Limitations of current meta-analyses should be noted. Due to the limited evidence available, previous meta-analysis data extracted from retrospective studies [46] and even case series [47], which might bring significant bias for the overall analysis. Most comparisons included only 1 or 2 studies due to the small number of clinical trials pooled for meta-analysis. More RCTs are responsible for the evaluation of the efficacy of PRP injection on pain relief and function improvement compared to HA injection. Our meta-analysis included 20 RCTs to investigate the efficacy of PRP injection on pain relief and function recovery compared with HA injection in patients with KOA. In short-term period postinjection (no more than 3 months), PRP injection resulted in better WOMAC function score at 1 month and WOMAC stiffness function score, stiffness score, and IKDC at 3 months compared with HA injection. PRP injection and HA injection had similar effects with respect to the WOMAC pain scores, WOMAC total scores, and VAS scores at 1 month and 3 months. And also, the patients with PRP injection showed similar effects in IKDC and EQ-VAS scores at 2 months, and KOOS at 3 months. In long-term period postinjection (no less than 6 months), we found that better clinical results were achieved in the PRP injection group compared with the HA injection group in terms of WOMAC pain, function, stiffness, and total scores and VAS scores at 6 months and 12 months. Moreover, the patients with PRP treatment showed better performance with respect to IKDC at 6 months and EQ-VAS at 12 months. Nevertheless, there was no significant difference between groups in terms of bias for the overall analysis. Most comparisons included only 1 or 2 studies due to the small number of clinical trials pooled for meta-analysis. More RCTs are responsible for the evaluation of the efficacy of PRP injection on pain relief and function improvement compared with HA injection.

Another issue that affects the effects of PRP is the leukocyte concentration in PRP composition, which may contain more proinflammatory cytokines and be detrimental to cartilage repair. Subgroup analyses were used to identify the potential heterogeneity of the results. A study conducted by Riboh et al. [48] compared LP-PRP and LR-PRP in the treatment of KOA and found that LP-PRP injections resulted in significantly improved WOMAC scores compared with HA or placebo. These results were consistent with our subgroup findings.

Vilchez-Cavazos et al. [45] conducted a meta-analysis and revealed that single injection was as effective as multiple PRP injections in pain improvement; however, multiple injections seemed more effective in joint functionality than a single injection at 6 months. Similarly, most of these results were also consistent in different PRP spinning approaches, whether with an activator use and PRP species (fresh or frozen), which suggested that these factors might have little influence on the efficacy of PRP.

### Limitations

Some limitations in the current study should be interpreted. Firstly, the main limitation is that most overall analyses are accompanied with high heterogeneity. The high heterogeneity among pooled results has weakened the persuasion of the conclusion. Although we tried to compensate for methodological deficiencies by performing stratified analyses, some results remained inconclusive since several reports lacked the documentation of the key factors. Secondly, although 19 RCTs were included in this study, some indexes such as WOMAC function and stiffness score at 1 month, IKDC at 3 months, EQ-VAS at 2 months, and KOOS at 3 months were analyzed by the data extracted from only two studies. Moreover, similar to the previous meta-analysis studies, we evaluated the efficacy between PRP and HA within 1 year on the account of limited follow-up. Some RCTs explored the long-term follow-up (52 weeks by Buendia-Lopez et al. [23] and 5 years by Di Martino et al. [14]). However, we were unable to pool the long-term results from the limited data. Thirdly, almost all included RCTs used subjective questionnaires to deduce the treatment effects. Objective findings such as magnetic resonance and ultrasound seem to be needed in the efficacy evaluation. Finally, the administration of PRP injection was varied in the included RCTs. The present study failed to recommend the optimal administration dosage and interval because of the insufficient data. Therefore, more well-designed RCTs with long-term follow-up are still necessary.

## Conclusion

Intra-articular PRP injection appeared to be more efficacious than HA injection for the treatment of KOA in terms of short-term functional recovery. Moreover, PRP injection was superior to HA injection in terms of long-term pain relief and function improvement. In addition, PRP injection did not increase the risk of adverse events when compared with HA injection. Additional RCTs are needed to identify the optimal doses and intervals of PRP and HA.

## Supplementary information

**Additional file 1.** Search strategies in PubMed database.

**Additional file 2.** PRISMA 2009 Flow Diagram.

## Data Availability

We state that the data will not be shared since all the raw data are present in the figures included in the article.

## Abbreviations

PRP: Platelet-rich plasma
HA: Hyaluronic acid
KOA: Knee osteoarthritis
WOMAC: Western Ontario and McMaster Universities Arthritis Index
IKDC: International Knee Documentation Committee
KOOS: Knee Injury and Osteoarthritis Outcome Score
ACR: American College of Rheumatology
PRISMA: Preferred Reporting Items for Systematic Reviews and Meta-analysis
MeSH: Medical Subject Headings
SMD: Standard mean difference
CI: Confidence interval
RRs: Risk ratios
