# Supplementary material for: Platelet-rich plasma versus hyaluronic acid in the treatment of knee osteoarthritis: a meta-analysis
Source: J Orthop Surg Res. 2020 Sep 11;15:403. doi: 10.1186/s13018-020-01919-9 (PMC7488405; doi:10.1186/s13018-020-01919-9)
Supplement: Supplementary file 1 — Additional file 1. Search strategies in PubMed database. [file 13018_2020_1919_MOESM1_ESM.docx]

Supplement S1: Search strategies in PubMed database.

((((((("Osteoarthritis, Knee"[Mesh]) OR Osteoarthritis of the Knee) OR Osteoarthritis of Knee) OR Knee Osteoarthritis) OR Knee Osteoarthritides)) AND ((((((((((((((("Hyaluronic Acid"[Mesh]) OR Hyaluronic Acid) OR Healon) OR Amvisc) OR Hyaluronate Sodium) OR Hyaluronate, Sodium) OR Sodium Hyaluronate) OR Luronit) OR Hyvisc) OR Hyaluronan) OR Etamucine) OR Biolon) OR Vitrax, Amo) OR Amo Vitrax) OR Acid, Hyaluronic)) AND (((("Platelet-Rich Plasma"[Mesh]) OR Platelet-Rich Fibrin) OR Platelet Rich Plasma) OR Plasma, Platelet-Rich)
