# Supplementary figures and images for: Platelet-rich plasma versus hyaluronic acid in the treatment of knee osteoarthritis: a meta-analysis
Source: J Orthop Surg Res. 2020 Sep 11;15:403. doi: 10.1186/s13018-020-01919-9 (PMC7488405; doi:10.1186/s13018-020-01919-9)

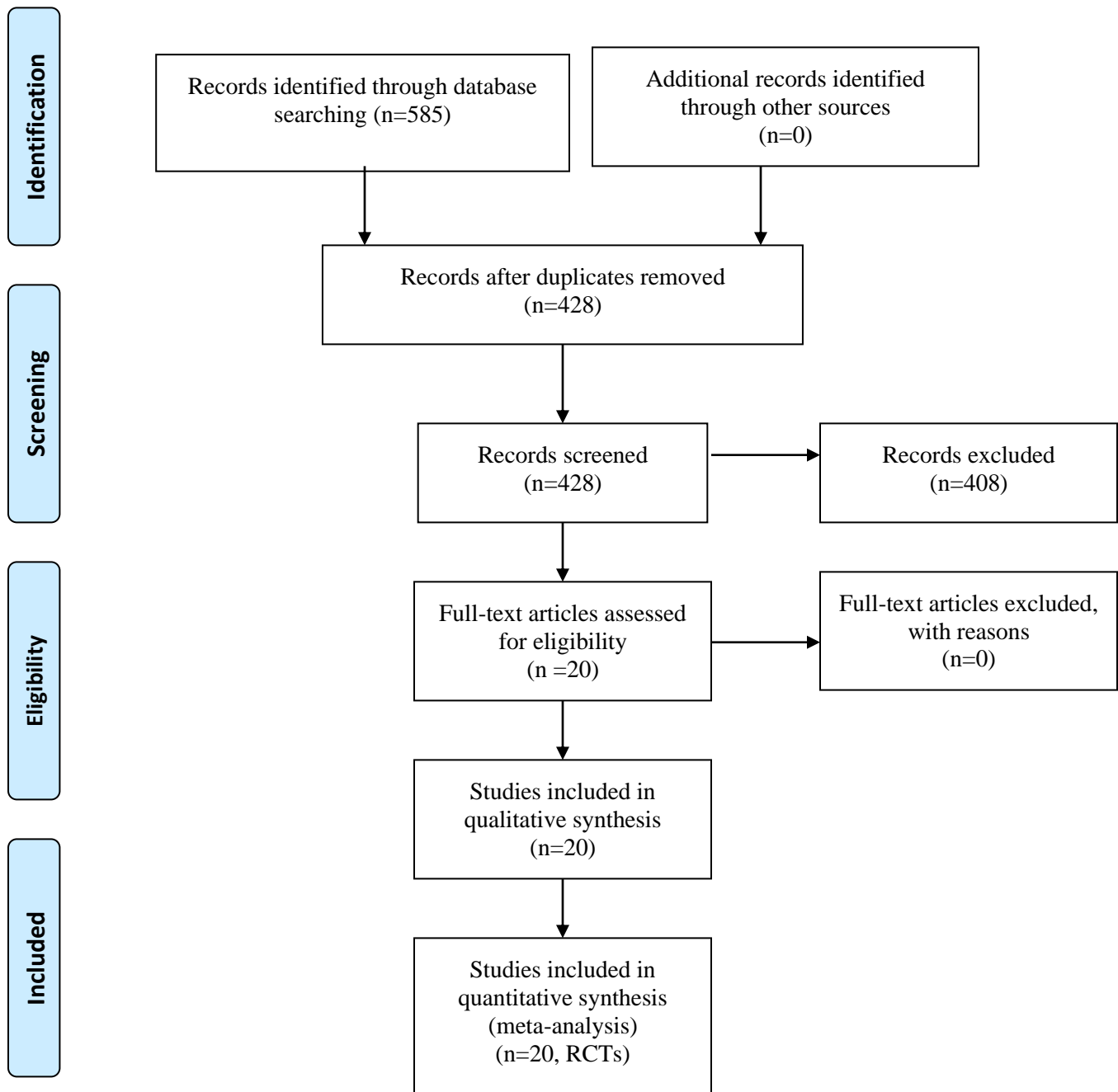

Supplement: Supplementary file 2 — Additional file 2. PRISMA 2009 Flow Diagram. [file 13018_2020_1919_MOESM2_ESM.pdf]
